# One size doesn’t fit all: exploring the influence of body size, age, and sex on right ventricle size measurements

**DOI:** 10.1186/s13089-025-00407-7

**Published:** 2025-02-24

**Authors:** Yun Wang, Christopher G. Scott, Garvan C. Kane, Sorin V. Pislaru, Jared G. Bird, Patricia A. Pellikka, Vidhu Anand

**Affiliations:** 1 Department of Internal Medicine, Mayo Clinic, 200 1st Street SW, Rochester, MN USA; 2 Department of Quantitative Health Sciences, Mayo Clinic, 200 1st Street SW, Rochester, MN USA; 3 Department of Cardiovascular Medicine, Mayo Clinic, 200 1st Street SW, Rochester, MN USA; 4 Department of Cardiovascular Medicine, Mayo Clinic Health System Northwest Wisconsin, 1400 Bellinger Street, Eau Claire, WI USA

**Keywords:** Right ventricle size, Dimensions, Sex differences, Body surface area

## Abstract

**Background:**

The assessment of right ventricular (RV) size is an important part of 2-dimensional transthoracic echocardiography. Current chamber quantification guidelines provide reference values as unindexed numbers, similar for men and women. We sought to evaluate normal ranges of RV dimensions based on age, sex, body surface area (BSA), and height.

Consecutive patients with “normal echocardiogram” between January 2011 and August 2022 at our center were retrospectively included. RV dimensions including diameter at the base and mid-ventricle level, and base-to-apex length were measured.

**Results:**

Of 1389 patients (median 43 years, 53% female) with all three measurements available, the median RV measurements, both unindexed and indexed to BSA, were: basal diameter 35.0 mm (31.0–39.0) and 18.4 mm/m^2^ (16.5–20.3); mid diameter 28.0 mm and 14.8 mm/m^2^ (13.1–16.6); RV length 73.0 mm (67.0–78.0) and 37.6 mm/m^2^ (34.9–40.9). RV dimensions were larger in men than women across all age groups but similar when indexed to BSA (for basal and mid dimensions). RV length was best indexed to height. Our indexed normal values by age and sex were similar to World Alliance Societies of Echocardiography (WASE) cohort.

**Conclusions:**

RV measurements should be indexed to BSA, considering sex and age, to determine RV size and enlargement.

**Supplementary Information:**

The online version contains supplementary material available at 10.1186/s13089-025-00407-7.

## Introduction

The assessment of right ventricular (RV) size is an important part of comprehensive transthoracic echocardiography. Although RV size is best assessed using 3-Dimensional (3-D) volumetric assessment, it is often not feasible due to lack of 3-D software or sonographer experience, limited study time and suboptimal imaging windows for adequate 3-D image acquisition. Therefore, RV size is frequently assessed using 2-Dimensional (2-D) linear dimensions: diameter at the base and mid ventricle level, and base-to-apex length. The current reference normal values in chamber quantification guidelines are provided as unindexed numbers and similar for men and women [1]. Multiple studies of 3-D echocardiography and cardiac magnetic imaging have shown that RV size parameters vary with body size, sex, age, and ethnicity [2–4]. These variations were also seen in 2-D echocardiography derived RV linear dimensions in the Normal Reference Ranges for Echocardiography (NORRE) and the World Alliance Societies of Echocardiography (WASE) study cohorts [4–6]. We sought to evaluate the normal ranges of RV linear dimensions based on age, sex, body surface area (BSA), height and validate the findings of WASE study [6] using patients with normal echocardiogram at our institution.

## Methods

Consecutive patients with “normal echocardiogram” noted in the final impressions by an experienced Level 3 trained echocardiographer and all measurements and findings suggesting normal cardiac exam, between January 2011 and August 2022 were retrospectively included. The RV size was measured in the apical RV focused view by a trained sonographer or retrospectively by a resident (YW) with training in measuring the same. Our institutional Echocardiography Laboratory complies with the American Society of Echocardiography (ASE) and the Intersocietal Accreditation Commission (IAC) Standards and Guidelines for Adult Echocardiography Accreditation. In the rare cases of multiple normal echocardiograms, the first one was included for the analysis. Data are presented as median (IQR). The groups were compared using analysis of variance and unpaired t-test. Trends across age group were tested using linear regression with age group treated as an ordinal variable. All analyses were performed in SAS and p < 0.05 was considered statistically significant. The normal ranges were defined at upper and lower limits using 5th and 95th percentile. DuBois formula [7] was used for BSA calculation.

## Results

The feasibility of individual RV measurements was as follows: basal dimension, 94.4%; mid dimension, 94.4%; and length, 94.0%. A total of 1389 patients who had all three measurements available were included in the final analyses. The median age was 43 years (31, 55), 53% were females. The distribution of patients by baseline characteristics and echocardiographic variables are presented in Supplemental Table 1. Among these patients, 266 (19%) had hypertension, 77 (6%) had diabetes, 62 (4%) had chronic obstructive pulmonary disease and 27 (2%) had coronary artery disease, but these conditions were either mild or controlled and didn’t result in cardiac damage (as noted on echocardiography) in these patients. All patients had ejection fraction of > 55%, normal diastolic function, and no significant valvular disease and normal reported RV size and function. The subjects were distributed among subgroups according to age and sex: 18–39 years (285 men, 297 women), 40–59 years (264 men, 284 women) and ≤ 60 years (102 men and 157 women). For the entire cohort, the median RV measurements, both unindexed and indexed to BSA, were as follows: basal diameter at 35.0 mm (range 31.0–39.0) and 18.4 mm/m^2^ (range 16.5–20.3); mid diameter at 28.0 mm and 14.8 mm/m^2^ (range 13.1–16.6); RV length at 73.0 mm (range 67.0–78.0) and 37.6 mm/m^2^ (range 34.9–40.9).

**Table 1:** RV linear dimensions presented in men and women, indexed by height (allometric indexing)

Parameters	All	Men	Women
RV mid/Height, mm/m	16.5 (14.6, 18.4)	17.1 (15.2, 18.8)	15.9 (14.1, 17.8)
RV mid/Height, mm/m^2.13^	9.0 (7.9, 10.0)	8.9 (7.8, 9.9)	9.1 (8.1, 10.3)
RV mid/Height, mm/m^1.7^	11.3 (10.0, 12.6)	11.4 (10.0, 12.6)	11.2 (10.0, 12.7)
RV basal/Height, mm/m	20.4 (18.5, 22.3)	21.0 (19.1, 22.9)	19.9 (18.1, 21.9)
RV basal/Height, mm/ m^2.13^	11.1 (10.0, 12.4)	10.8 (9.8, 12.0)	11.4 (10.3, 12.7)
RV basal/Height, mm/m^1.7^	14.0 (12.7, 15.4)	13.9 (12.6, 15.3)	14.1 (12.8, 15.6)
RV length/Height, mm/m	42.2 (39.6, 44.6)	42.5 (40.6, 44.8)	41.8 (38.9, 44.4)
RV length/Height, mm/m^2.13^	22.9 (21.2, 24.8)	22.0 (20.6, 23.5)	23.8 (22.0, 25.7)
RV length/Height, mm/m^1.7^	28.9 (27.0, 30.9)	28.3 (26.6, 29.9)	29.6 (27.3, 31.6)

Data are expressed as median (IQR)

*RV mid*, RV mid-ventricle diameter; *RV basal*, RV basal diameter; *RV length*, RV apex-base length

**Fig. 1:**
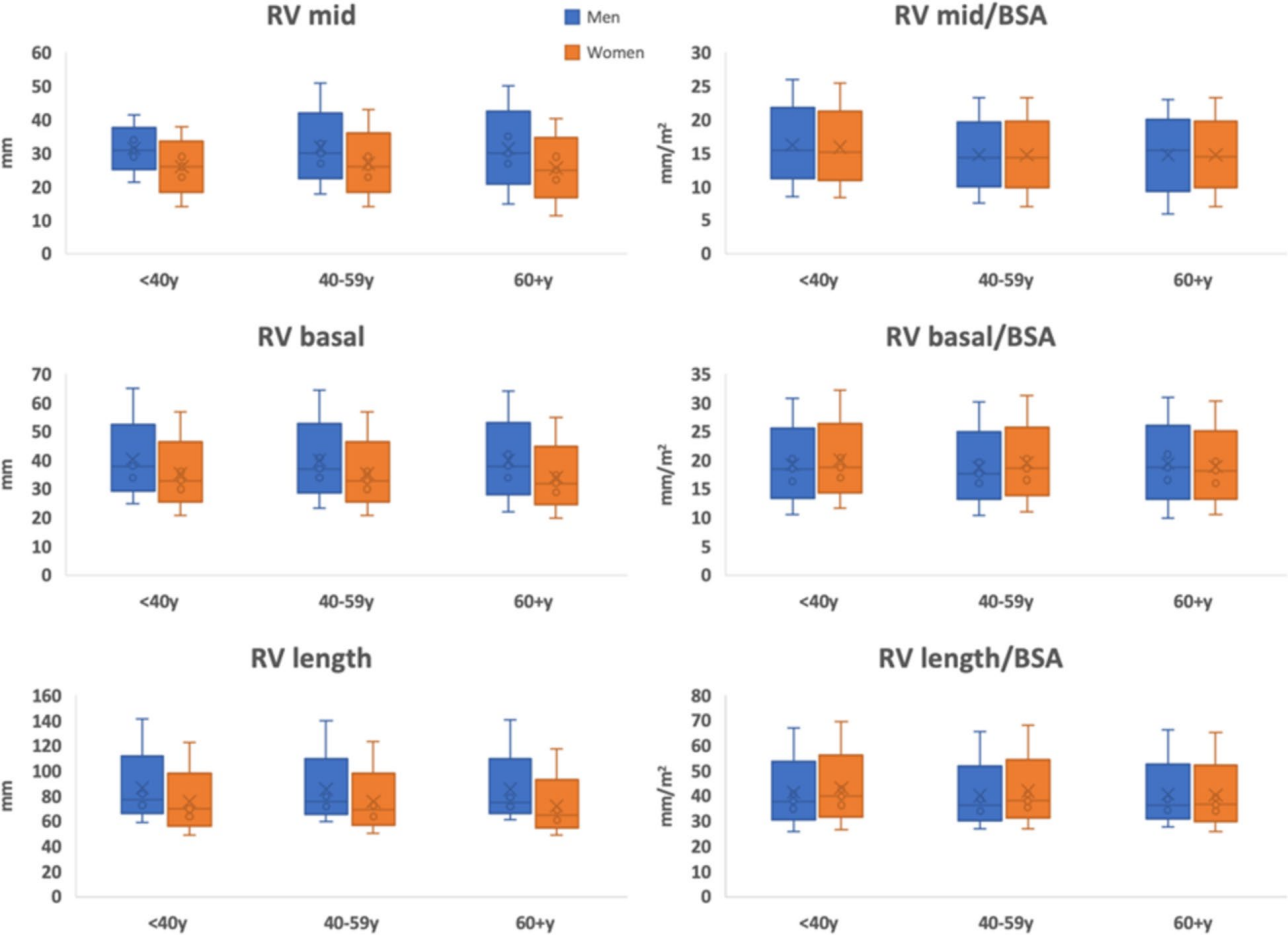
Right ventricle (RV) dimensions according to age and sex, unindexed and indexed to body surface area. *RV mid* RV mid-ventricle diameter, *RV basal* RV basal dimension, *RV length* RV base-apex dimensión

The age and sex-stratified dimensions at basal and mid RV and base-apex length are presented in Fig. 1. All three linear dimensions were larger in men than women. When indexed to BSA, there were no significant differences between men and women for RV basal and mid dimensions and RV length in all age groups (Supplemental Table 1). Similar results were obtained after excluding the patients with comorbidities such as chronic obstructive pulmonary disease and/or coronary artery disease (n = 86) (Supplemental Table 2). The RV length decreased, and mid dimension increased with age (p < 0.001), but there were no significant differences in basal diameter with age (p = 0.32). Indexing to height yielded similar results but the reference normal were different and RV dimensions overall remained larger in men while indexing to height. Allometric indexing (height^b) was evaluated and our data yielded an allometric index around 1(b) for all three RV dimensions suggesting relatively linear relationships. Common allometric exponents of 1.7 and 2.3 for height were used and all dimensions were larger in women compared with men when indexed to these values (Table 1 and supplemental Table 3 for patients excluding chronic obstructive pulmonary disease and/or coronary artery disease). RV mid and basal dimensions are best indexed to an allometric exponent of 1.7 and RV length remains best indexed to height itself (b = 1). Table 2 shows our established normal values compared with those established from WASE study and current guidelines. Our indexed normal values stratified by age and sex were similar to the WASE cohort. Larger unindexed basal and mid diameters were noted in our cohort when compared to the guideline normal, but these were similar to the WASE cohort.

**Table 2:** Normal values for RV linear dimensions

	Current study			Addetia et al. (WASE study)			2015 Guideline
Parameters	All	Men	Women	All	Men	Women	All
	5th–95th	5th–95th	5th–95th	2 SD	2 SD	2 SD	2 SD
RV mid, mm	20.0–37.0	22.0–39.0	20.0–34.0	16.3–38.6	17.7–40.7	15.5–34.4	19–35
RV mid/BSA, mm/m^2^	10.6–19.2	10.8–19.2	10.5–19.3	9.5–20.9	9.4–21.0	11.4–17.4	
RV basal, mm	27.0–44.0	28.0–47.0	25.0–41.0	23.2–44.2	24.5–45.4	22.4–39.8	25–41
RV basal/BSA, mm/m^2^	13.8–23.0	13.6–22.7	13.8–23.3	13.6–24.5	13.5–24.4	16.5–20.1	
RV length, mm	59.0–85.0	66.0–87.0	57.0–81.0	57.5–91.8	61.1–94.0	56.2–83.7	59–83
RV length/BSA, mm/m^2^	30.9–45.3	31.1–44.4	30.7–45.9	32.4–52.5	32.2–51.4	41.5–42.3	

5th to 95th, 5th to 95th percentile; *SD* standard deviation; *RV* mid RV mid-ventricle diameter; *RV basal* RV basal diameter; *RV length* RV base-apex length

## Discussion

Our study has several important findings. We have established the normal values for 2-D RV basal and mid diameter and length, indexed to BSA and stratified by age and sex, in a large cohort of patients seen at our institution. We found these reference normal values to be similar to the WASE cohort [6]. The RV dimensions were larger for men when compared to women in all age groups but were similar when indexed to BSA (for basal and mid dimensions). RV length is best indexed to height. We found that RV mid dimension increased with age and length decreased with age (also observed in WASE cohort). Our study highlights that one size (reference for normal values) doesn’t fit all the right ventricles and measurements should be indexed to body surface area and consideration should be given to sex and age of the patient to ascertain the RV size and enlargement. When BSA is unavailable, height and allometric exponent of height can be used for indexing, keeping in mind that the normal reference values differ for height, and RV dimensions remain slightly higher for men even after indexing to height although the relationship reverses when using allometric exponent. The limitations of the study include retrospective study design which allows measurements only on the available RV focused views and limited representation of different ethnicities and races due to geographical location of our institution and a referral center. Although a small portion of our patients had medical comorbidities, they did not have evidence of cardiac damage. These patients are likely more representatitve of a real world cohort. Due to unavailability of chest computed tomography in most patients, the impact of chest wall shape and diameter on RV size could not be evaluated. [8]

In summary, RV size is dependent on body size, and the assessment should take into account factors such as sex and age of the patient.

## Supplementary Information

Additional file 1.

## Data Availability

The datasets used in the current study can be made available from the corresponding author upon reasonable request, and after institutional approval.
