# Supplementary material for: One size doesn’t fit all: exploring the influence of body size, age, and sex on right ventricle size measurements
Source: Ultrasound J. 2025 Feb 24;17:14. doi: 10.1186/s13089-025-00407-7 (PMC11850662; doi:10.1186/s13089-025-00407-7)
Supplement: Supplementary file 1 — Additional file 1. [file 13089_2025_407_MOESM1_ESM.docx]

| Supplemental Table 1. Characteristics of the study population | | | | |
| --- | --- | --- | --- | --- |
|  | All  (n = 1389) | Men  (n = 651) | Women  (n = 738) | P-value |
| Characteristics |  |  |  |  |
| Age, y | 43 (31, 55) | 42 (31, 54) | 44 (32, 57) | 0.06 |
| Age groups |  |  |  |  |
| 18-39 y | 582 (42%) | 285 (44%) | 297 (40%) |  |
| 40-59 y | 548 (39%) | 264 (40%) | 284 (39%) |  |
| ≥ 60 y | 259 (19%) | 102 (16%) | 157 (31%) |  |
| BMI, kg/m^2^ | 26.5 (23.2, 30.7) | 26.9 (24.0, 30.7) | 26.1 (22.4, 30.8) | 0.018 |
| BSA, m^2^ | 1.9 (1.7, 2.1) | 2.1 (1.9, 2.2) | 1.8 (1.7, 1.9) | <.001 |
| Height, m | 1.7 (1.6, 1.8) | 1.8 (1.7, 1.8) | 1.6 (1.6, 1.7) | <.001 |
| HTN, n (%) | 266 (19%) | 145 (22%) | 121 (16%) | 0.006 |
| CAD, n (%) | 27 (2%) | 18 (3%) | 9 (1%) | 0.038 |
| DM, n (%) | 77 (6%) | 39 (6%) | 38 (5%) | 0.50 |
| HLD, n (%) | 360 (26%) | 187 (29%) | 173 (23%) | 0.026 |
| COPD, n (%) | 62 (4%) | 29 (4%) | 33 (4%) | 0.98 |
| Afib, n (%) | 81 (6%) | 55 (8%) | 26 (4%) | <.001 |
| CKD, n (%) | 29 (2%) | 20 (3%) | 9 (1%) | 0.016 |
| Echocardiographic parameters |  |  |  |  |
| RV mid, mm | 28 (24, 32) | 31.0 (27.0, 34.0) | 26.0 (23.0, 29.0) | <.001 |
| RV mid/BSA,  mm/m^2^ | 14.8 (13.1, 16.6) | 14.9 (13.2, 16.7) | 14.8 (13.0, 16.5) | 0.29 |
| RV basal, mm | 35.0 (31.0, 39.0) | 37.0 (34.0, 41.0) | 33.0 (30.0, 36.0) | <.001 |
| RV basal/BSA,  mm/m^2^ | 18.4 (16.5, 20.3) | 18.2 (16.2, 20.2) | 18.6 (16.7, 20.3) | 0.06 |
| RV length, mm | 73.0 (67.0, 78.0) | 76.0 (72.0, 80.0) | 69.0 (63.0, 73.0) | <.001 |
| RV length/BSA,  mm/m^2^ | 37.6 (34.9, 40.9) | 36.9 (34.6, 39.8) | 38.5 (35.5, 41.5) | <.001 |
| Data are expressed in median (IQR) or number (percentage)  Abbreviations: *BMI*, body mass index; *BSA*, body surface area; *HTN*, hypertension; *CAD*, coronary artery disease; *DM*, diabetes mellitus; *HLD*, hyperlipidemia; *COPD*, chronic obstructive pulmonary disease; *Afib*, atrial fibrillation; *CKD*, chronic kidney disease; *RV mid*, RV mid-ventricle diameter; *RV basal*, RV basal diameter; *RV length*, RV apex-base length. | | | | |

Supplemental Table 2. Normal values of RV dimensions (5^th^ to 95^th^ percentile) in all cohort, men and women – after exclusion of 86 subjects with COPD and/or CAD

| Parameters | All | Men | Women |
| --- | --- | --- | --- |
|  | 5^th^ to 95^th^ | 5^th^ to 95^th^ | 5^th^ to 95^th^ |
| RV mid, mm | 20.0 to 37.0 | 23.0 to 39.0 | 20.0 to 34.0 |
| RV mid/BSA  mm/m^2^ | 10.7 to 19.3 | 10.9 to 19.2 | 10.5 to 19.3 |
| RV basal, mm | 27.0 to 44.0 | 29.0 to 47.0 | 25.0 to 41.0 |
| RV basal /BSA  mm/m^2^ | 13.8 to 23.0 | 13.8 to 22.6 | 13.8 to 23.3 |
| RV length, mm | 59.0 to 85.0 | 66.0 to 87.0 | 57.0 to 81.0 |
| RV length/BSA  mm/m^2^ | 31.1 to 45.4 | 31.3 to 44.3 | 31.0 to 46.0 |

Supplemental Table 3. RV linear dimensions presented in men and women, indexed by height (allometric indexing) – after exclusion of 86 subjects with COPD and/or CAD

| Parameters | All | Men | Women |
| --- | --- | --- | --- |
| RV mid/Height,  mm/m | 16.5 (14.6, 18.4) | 17.1 (15.3, 18.9) | 15.9 (14.1, 17.8) |
| RV mid/Height,  mm/m^2.13^ | 8.9 (7.9, 10.0) | 8.9 (7.8, 9.9) | 9.1 (8.1, 10.3) |
| RV mid/Height,  mm/ m^1.7^ | 11.3 (10.0, 12.6) | 11.4 (10.1, 12.6) | 11.2 (10.0, 12.6) |
| RV basal/Height,  mm/m | 20.5 (18.6, 22.4) | 21.0 (19.1, 22.9) | 19.9 (18.1, 22.0) |
| RV basal/Height,  mm/ m^2.13^ | 11.1 (10.1, 12.4) | 10.8 (9.8, 12.0) | 11.4 (10.3, 12.7) |
| RV basal/Height,  mm/ m^1.7^ | 14.0 (12.8, 15.5) | 13.9 (12.7, 15.4) | 14.1 (12.8, 15.6) |
| RV length/Height,  mm/m | 42.2 (39.7, 44.7) | 42.6 (40.7, 44.8) | 41.8 (39.1, 44.5) |
| RV length/Height,  mm/ m^2.13^ | 22.9 (21.2, 24.8) | 22.1 (20.6, 23.5) | 23.8 (22.0, 25.7) |
| RV length/Height,  mm/ m^1.7^ | 28.9 (27.0, 30.9) | 28.3 (26.7, 30.0) | 29.6 (27.3, 31.6) |

Data are expressed as median (IQR)

*RV mid*, RV mid-ventricle diameter; *RV basal*, RV basal diameter; *RV length*, RV apex-base length.
